# Candidate-gene studies related to drug pharmacokinetics, pharmacodynamics, safety, and efficacy in the era of extensive technological developments: could we empower them by more efficient implementation of established epidemiological concepts?

**DOI:** 10.3325/cmj.2024.65.457

**Published:** 2024-10

**Authors:** Vladimir Trkulja

**Affiliations:** Department of Pharmacology, Zagreb University School of Medicine, Zagreb, Croatia; *vladimir.trkulja@mef.hr*

The ultimate goal of pharmacogenomics/pharmacogenetics (Pgx) research is to identify genetic traits that could help us decide – based on pre-emptive information (1) – about pharmacological treatment options most likely to be successful and acceptably tolerable in each individual patient given her or his medical condition. The past two decades have witnessed considerable advancement in accumulated substantive knowledge and in research methods (technological and conceptual) (1-6). Considering the latter, it has been suggested that traditional candidate-gene studies might have become outdated having in mind their numerous limitations (7). It is true that candidate-gene studies might be of little value in complex settings (multifactorial, polygenic, with many gene-environment interactions) such as the occurrence of diseases (8), and psychological and behavioral traits (9). However, in pharmacogenetics settings (drug pharmacokinetics and effects), which are generally less complex and mechanistically better understood, candidate-gene studies can still provide reliable and valuable insights, if they are adequately planned and conducted (10). A recent systematic elaboration offers a detailed methodological roadmap for the conduct of meaningful candidate-gene Pgx studies (10). The present comments intend to provide supplementary views in part from the standpoint of the concept of causality (11) widely applied in epidemiology (12), with some use of elementary directed acyclic graphs (DAGs) (13).

## We are interested in causes and effects

**Figure 1:**
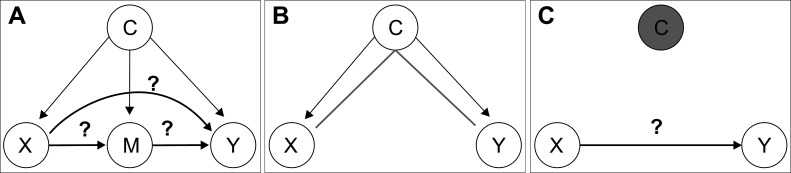
A basic situation in which a confounder (**C**) interferes (introduces bias) with identification and quantification of a causal effect of a cause (**X**) on the outcome of interest (**Y**). The schematics are simplified directed acyclic graphs (DAGs). (**A**) The basic setting: the intention is to assess whether there is a causal effect of X on Y and quantify it. The investigated hypothesized (indicated by “?”) causal path is depicted by thick black arrows. They start at X and are directed toward Y. The “total effect” could be in part mediated by some other factor (mediator, M, where X causes M and M causes Y; there could be more than one mediator, in a serial sequence, or parallel, or any combination of those), or could go without a mediator (directly). Confounders (**C**) are variables (one or more; thin black arrows starting from C and directed to other elements) that cause both X (or descendants of X, eg, M) and Y, or are non-causally associated with X and cause Y (see text). (**B**) A situation in which there is no causal effect of X on Y (no thick black arrows directed from X to Y), but there is an open non-causal (“backdoor”) path between them depicted by gray lines: when not accounting for C, X and Y will be statistically associated via C. Moreover, even if there is a causal effect of X on Y, if C is not conditioned on, Y has two causes, X and C. Hence, the change in the value of Y observed after “switching” X from one value to another (eg, from 0 to 1) may not be due only to this fact. (**C**) A situation appropriate for evaluating (presumed – indicated by “?”) causal effect of X on Y: C is conditioned upon (gray circle, no arrows starting from it); hence, the non-causal paths via C (any number of Cs) are blocked: if a statistical association between X and Y exists – it results from a causal effect of X on Y.

The focus of a Pgx candidate-gene study is to evaluate whether a particular variant (eg, polymorphism, genotype, haplotype, diplotype, a genetic variant), ie, variable X, reliably predicts a drug’s pharmacokinetics, efficacy/safety, namely, the future event Y. In some situations, the relationship between X and Y does not need to be causal in order for X to well predict Y. Assume that daily, without exception, bus X leaves the terminal at 08:00 am and bus Y leaves it at 08:04 am. Seeing bus X leaving the terminal informs us perfectly about the imminent events regarding bus Y, but there is no causal relationship between the two events. Their perfect association is due to their common cause (C, a confounder) – the timetable; by this fact, X and Y are non-causally associated (Figure 1A, B) (11-14). Figure 1 indicates a causal effect of C on both X and Y, but the confounder does not need to be causal to X (the arrow from C to X may also indicate a non-causal association). The most intuitive definition of a confounder is that it is a variable that opens a non-causal (“backdoor”) path between X and Y (11-13). However, in such a setting the prediction of Y by X is not driven by X – it is driven by C: for a successful prediction, all three elements have to be in place. One may conceive a situation in which we have X and Y but we do not have C (eg, buses leave as their drivers see fit) – the association between X and Y is lost. Consequently, finding a non-causal association between X and Y (driven by C) in candidate-gene Pgx studies is not of much use: C could be present in one, but not in some other sample from the target population. This obstructs the efforts to define principles generalizable to the target population and its individual members. When the relationship between X and Y is causal, ie, when a change in X (eg, from 0 to 1) causes a change in Y, then the prediction of the future value of Y is more likely to hold in any sample from the target population. In reverse, repeatedly observed associations from different samples strengthen the probability that the relationship of interest is indeed a causal one (15). Therefore, although we commonly use the term “candidate-gene association” we are interested in causal effects of a certain “treatment” or “exposure” (a genetic variant) on the outcome: drug pharmacokinetics, efficacy, or safety. The interpretability of the candidate-gene Pgx studies largely depends on the biological plausibility of the setting. Biological plausibility implies a mechanistic rationale for the evaluated relationship – and this implies causality. Hence, the core value of any such study is in the extent to which it manages to estimate a true causal effect (11-15).

**Figure 2:**
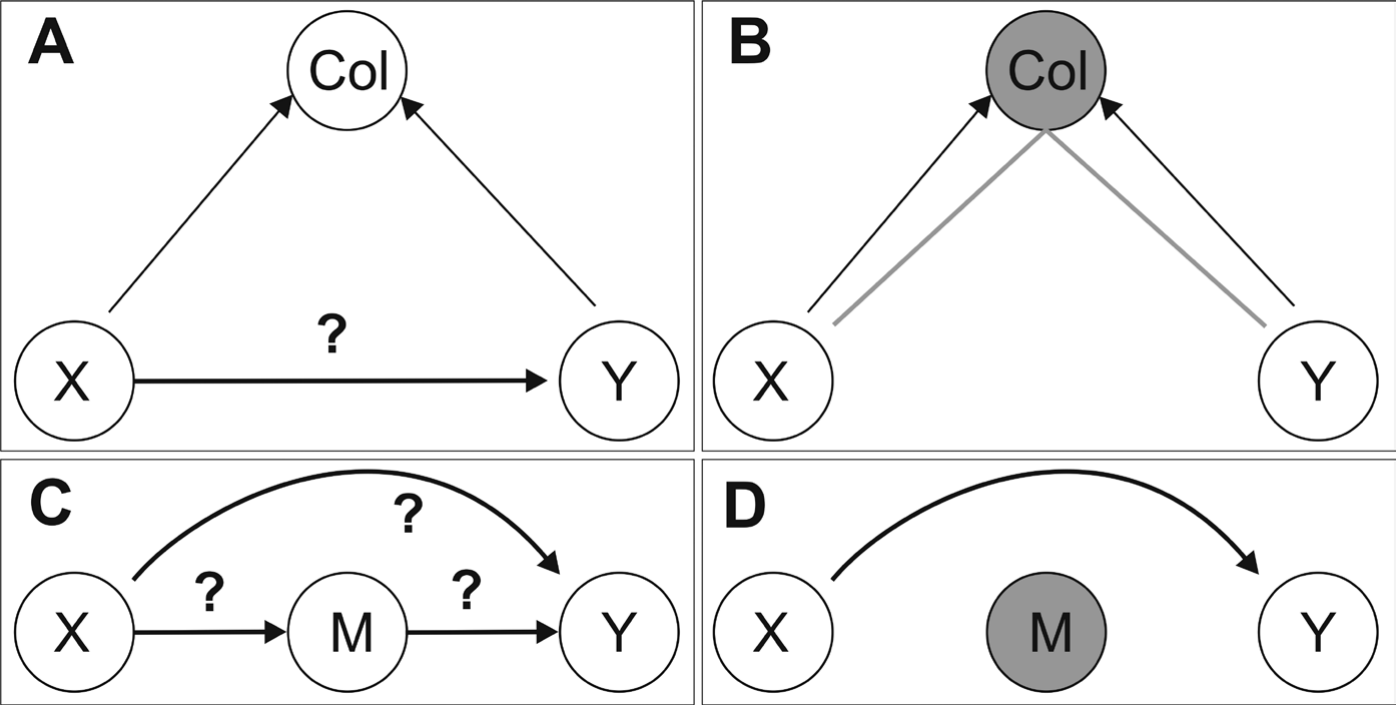
Simplified directed acyclic graphs (DAGs) illustrate how confusing a collider (Col) (**A, B**) or a mediator (**M**) (**C, D**) for a confounder can bias the estimation of the causal effect of X on Y. (**A**) The intention is to estimate the hypothesized causal effect of X on Y (thick black arrow from X to Y, with “?”). For simplicity, the effect is only direct (no mediators). There are no confounders, no open non-casual paths between X and Y. However, another arrow starts from X and ends in a collider – X causes Col, but Col is not a mediator: there are no arrows starting from Col to Y. Instead, there is an arrow directed from Y to Col – Y also causes Col. Colliders are variables located “between” X and Y (“on the path between X and Y”), but they do not cause either of them, rather they are caused by X (or any descendant of X, eg, a mediator – here not shown for simplicity) and Y (or any descendant of Y). Based on these properties, colliders, if disregarded, are not points through which a non-causal link between X and Y could be established, they actually block it (this is symbolized by “incoming” arrows). Hence, the situation is suitable for the purpose. (**B**) If a Col is confused for a confounder and conditioned on, this introduces bias – it opens a non-causal path between X and Y (indicated by gray lines). Assume, for simplicity, binary X, Y, and Col. Assume that “switching” X from 0 to 1 causes Col to change from 0 to 1, and that “switching” Y from 0 to 1 causes the same, then by conditioning on a collider (gray circle), a non-causal path between X and Y is opened (indicated by gray lines): even if there is no causal effect of X on Y (no black arrows between X and Y), they will be statistically associated (non-causally) because values X = 1 and Y = 1 are “linked” by being “grouped” within Col = 1. (**C**) The intention is to evaluate the presumed causal effect of X on Y, the direct and the mediated (via a mediator, M) part (thick black arrows, with “?”). There are no non-causal paths open: the setting is ready for the purpose. (**D**) If a mediator is confused for a confounder, and conditioned on (gray circle), then the mediated part of the causal effect is blocked: only a direct effect could be identified. Conditioning on a mediator means that the relationship between X and Y is estimated separately in units with M = 0 and separately in units M = 1; hence, the causal effect of X that goes “through” M (“switching” X from 0 to 1 causes M to change from 0 to 1, and “switching” M from 0 to 1 causes Y to change from 0 to 1) is blocked.

Absolute or relative effect measures, or those in population (pharmacokinetics/pharmacodynamics) modeling studies (16), quantify statistical associations (11-15). They inform about a causal effect only when all non-causal paths between X and Y are blocked (Figure 1C) (11-15). In a well-conceived, conducted, and analyzed randomized experiment, this is achieved by study design and conduct – treated subjects and controls are exchangeable by the virtue of randomization (12,14), and the trial is free of other possible fallacies (17). Candidate-gene studies or population models that consider candidate-genes as covariates are not randomized trials (18). “Exposures” (variants) are “assigned” as a random draw of the genes of our parents (19), but this assignment may depend on the known or unknown characteristics that affect the outcome of interest since these could also be largely genetically determined and linked to the exposure of interest (18,19). Hence, there could be systematic and chance imbalances between the exposed and control subjects in one or more measured or unmeasured characteristics that affect the outcome. By the given imbalance, they open paths for non-causal X and Y association (Figure 1) (18). This problem is common to all non-randomized (observational) studies; hence, the same methods used to minimize it should be applied in candidate-gene studies – the objective is to attain a balance between exposed subjects and controls regarding such characteristics. However, unlike in a valid randomized experiment, in observational studies one can achieve such a balance only regarding the characteristics that are known and measured (conditional exchangeability) (12,14,17,18) – a confounder (or a set of confounders) may remain unmeasured. The estimation of a causal effect depends on the extent to which such unmeasured confounding has been minimized. Of course, this applies if the study is not burdened with other types of systematic errors imminent to any observational study (12-14,17,18), including those specific to the genetic setting, eg, population stratification bias (10). Therefore, as in any observational study, all observed effect measures should be assessed for their susceptibility to various potential biases. The simplest and most straightforward analysis of this type is the analysis of sensitivity to unmeasured confounding by, for example, determining the E-value (a measure of the strength of the hypothetical confounding effect needed to explain the observed effect) (20), or by correcting the observed estimates for a (hypothetical) confounding effect of a certain size (21). Care should be taken in the selection of variables to condition on: conditioning on confounders will block non-causal associations (Figure 1C); however, conditioning on colliders or mediators – when confused for confounders – will introduce bias either by opening non-causal paths (in the case of colliders) (Figure 2A, B) or by blocking the true causal paths (in the case of mediators) (Figure 2C,D) (11,13,14,22). Cross-sectional studies are generally inappropriate for the detection of causal effects, since the required temporal sequence is difficult or impossible to establish (the presumed cause should precede the consequence) (12). With candidate-gene Pgx studies in simpler settings, such as pharmacokinetics, this may not be a problem, and many candidate-gene pharmacokinetic studies are cross-sectional. However, in such studies, it might not be easy to distinguish between confounders/colliders/mediators – since all are present at the same time.

The essence of X as a “cause” is that by setting it from one value to another (formalized by the do-operator do[X]) a change is induced in the value of Y (11). This might be interpreted in a way that sees manipulability of X by an external intervention as a prerequisite for X to be designated as a cause. There have been extensive debates, particularly in the social sciences, whether non-manipulable (immutable) Xs, such as race, could be considered “causes” (23), and this may well be extended to a genetic variant in the focus of a candidate-gene Pgx study. There is, however, a theoretical background that supports the concept of “non-manipulable causes” (24,25). Strictly speaking, on the other hand, the investigated genetic variant is not the immediate cause of a drug-related outcome – the function of the encoded protein determined by the respective variant is the actual cause. The variant can affect the outcome exclusively through a change in the respective protein; hence, it meets one of the essential conditions of an instrumental variable (11,13), and such a view fits the concept of Mendelian randomization studies (18,19). There have been extensive debates on the applicability of the instrumental variable concept and respective data analysis methods in genetic studies of different types and in different settings (18,19,26) – a topic by far out of the present scope. In typical Pgx candidate-gene studies –conducted in relatively simple and mechanistically well-understood settings in which possible pleiotropy of genetically driven consequences is generally of a minor concern – it most likely does not matter whether the investigated genetic variant is viewed as an instrument or as a cause (where, then, the protein activity is a mediator, and X can only have a mediated effect on Y). In either case, one will contrast different levels of the variant regarding the outcome of interest. In both cases, the same potentially interfering factors (eg, other polymorphisms, protein inhibitors/inducers) should be considered as potential confounders of the instrument-outcome or “exposure/treatment”-outcome relationship (that is, the “instrumental variable” type of analysis will not resolve the problem of confounding).

**Figure 3:**
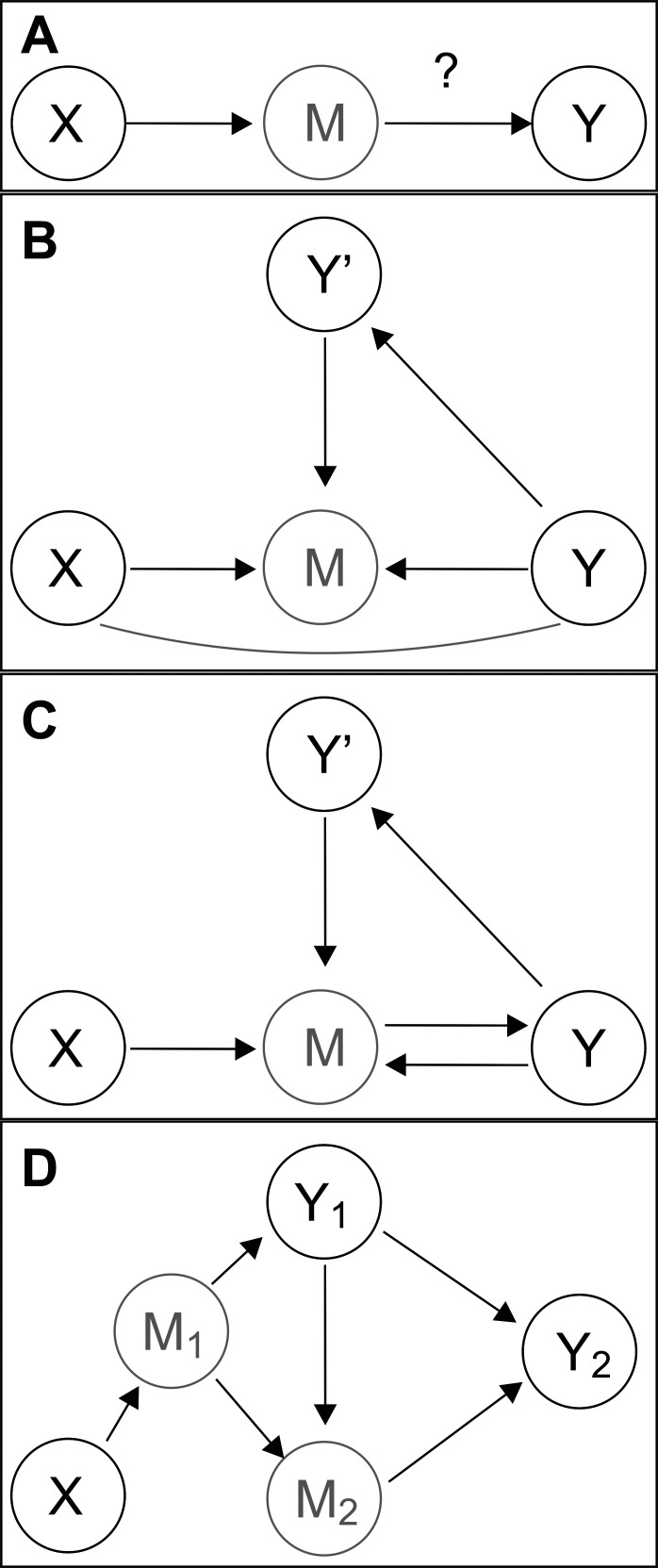
The problem of reverse/simultaneous causation. For simplicity, there is no confounding. (**A**) A directed acyclic graph depicts the setting: a genetic variant is considered a cause (**X**) and it is hypothesized (depicted by “?”) that it affects the outcome (**Y**), which is only possible through the effect on the (unmeasured) mediator (**M**) – protein function. (**B**) X affects M, but there is no causal effect of X on Y (no black arrow to connect them), yet a statistical association between X and Y is observed (gray line that connects them), since Y (or some of its descendants, Y’) causes M. (**C**) There are simultaneous effects – that is, consecutive effects in short subsequent time intervals – of M (a descendant of X) on Y, and of Y on M (directly or indirectly). (**D**) A possible scenario of reverse causation over time: X (a genetic variant, say, SNP) results in an altered protein function at time 1 (M1), which causes the outcome Y at time 1 (Y1); however, both M1 and Y1 cause the protein function at time 2 (M2), and then Y1 and M2 cause the outcome at time 2 (Y2).

If, in disagreement with the initial assumption, X does not cause Y, but Y causes X, X and Y will be statistically associated (12), and, considering the conceptual pretext, this can be misinterpreted as a causal effect of X on Y. Bi- and multidirectional causal effects and complex interplays between a range of elements and feedback loops are essential to all biological systems. Experiments allow one to detangle directions and sequences of causal effects, but in observational studies this might be tricky. Even in the case of non-manipulable Xs (such as a genetic variant), reverse or simultaneous causations (X-Y, Y-X) are possible (27). Assume that the intention is to estimate the effect of an SNP (X) on a drug-related outcome (Y) mediated through the consequence that the SNP has on the protein (eg, enzyme, transporter) (M) (Figure 3A). Even if no such effect exists, X and Y will be associated if Y affects M (Figure 3B). It is also possible that simultaneously (ie, in a sequence of adjacent short time intervals) X affects Y via M, and Y affects M (Figure 3C). An example may be that of lamotrigine, which induces its own metabolism (28); hence, over the first 2-3 weeks of treatment (until maximum induction has been achieved) it is impossible to conclude whether lamotrigine concentration at a certain point (Y) is due to some SNP (X) in the gene encoding the metabolizing enzyme or it is driven primarily by the effect of lamotrigine (Y) on the enzyme activity (M). Obviously, adequate timing of a study may help avoid the problem. This indicates that in longitudinal studies, the sequence of causal directions might be more readily adequately established (although it may not always be a simple task, Figure 3D), while in cross-sectional settings avoiding reverse causation might be problematic.

**Figure 4:**
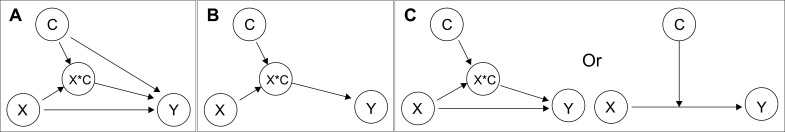
Interaction and effect modification (for simplicity, there is no confounding or mediation). There are no uniformly accepted directed acyclic graph (DAG) representations of these concepts, although several proposals have been made (30-32). The present DAGs mainly follow the concept presented in ref. 32. (**A**) Interaction: Y has two causes, X and C, which interact (mechanistically) (X*C) in their effects on Y: the joint effect (X*C) differs from the sum of the individual effects (X, C). (**B**) Sufficient cause concept: neither X nor C are sufficient to cause Y, but are necessary – when both are present (X*C), they cause Y. (**C**) Effect modification: Y has only one cause (**X**), but the effect is heterogeneous, ie, it differs at different levels of C.

The term “interaction” is common in genetic epidemiology, particularly in the concept of gene-environment interaction (29) in the occurrence of diseases. Typically, “interaction” is used in the sense of simultaneous causes of the outcomes of interest and their co-operation in the process. It is assumed that an outcome (Y) has – for simplicity - two causes (X and C); hence, a switch in either X or in C from 0 to 1 causes a change in the value of Y. If the value of Y with X = 1 and C = 1 equals the sum of its values when only X = 1 or only C = 1 – there is no interaction; if the value is greater than the sum of individual effects – there is a synergistic interaction; if the value is lower than the sum of individual effects – there is an antagonistic interaction (29). Figure 4A shows a DAG that indicates interaction (30-32). The term “interaction” is also employed within the concept of sufficient/component causes (29) of an outcome – simplistically, each of the two causes is needed, yet none is sufficient to cause Y, but when both are “in action,” Y occurs (Figure 4B). The present point, however, is that the term “interaction” is commonly used with a different process in mind – the effect modification, ie, effect heterogeneity (29). The effect of X on Y at one level of a third variable (C) differs from the effect of X on Y at another level of that variable (Figure 4C). If the effect of X on Y is heterogeneous: it might exist at one level of C but not at another one, or it might be more pronounced (vs less), or it might be in one direction at one, and in the opposite direction at the other level of C (in which case these could cancel out to result in a null overall effect) (29). The third variable (C) is an effect modifier (or a moderator) (29). In such a case, C has no effect on Y, but the effect of X differs at different levels of C (Figure 4C). However, Figure 4A and 4C are similar (only the arrow from C to Y is missing in 4C): typically, when there is an interaction, there is also effect modification (and *vice-versa*) but each is also possible “without” the other (29). If effect modification exists and is overlooked, the overall effect estimate will not be an accurate picture of reality since there will be several different effects of X in different population subsets. It is equally as important to demonstrate effect heterogeneity as it is to demonstrate effect homogeneity since both are relevant for clinical implementation of Pgx information.

A number of systematic errors can obstruct the identification and quantification of a causal effect, both in randomized experiments and in observational studies such as candidate-gene Pgx studies. Figures 1-4 simplistically illustrate the elementary (and probably the most common) ones. What is not obvious from these schematics is the fact that they may vary over time, ie, over different periods of time, causes, colliders, mediators, confounders, effect modification, and/or interaction and reverse causation might be present or absent (eg, in longitudinal or other prospective studies) (17).

## Small and large samples, small and large effects, and meta-analysis of candidate-gene Pgx studies

What is considered a “sufficiently large” sample in a certain study depends on many factors. Candidate-gene studies are specific in that some variants of interest are very rare. Even if the variant prevalence is 10%-20% (a “common variant”) and the total sample size appears “reasonable,” the absolute number of carriers in an individual study might be low. The consequence is the fragility of the observed outcomes. A recent study (33) investigated an SNP in the *ABCB1* gene (*c.1236C>T* [rs1128503]) and the risk of diarrhea and mucositis in patients with metastatic colorectal carcinoma treated with irinotecan. The study enrolled 307 patients, 107 (34.9%) with the CC, 145 (47.2%) with the CT, and 55 (17.9%) with the TT genotype. The incidence of “severe diarrhea” was 22/107 (20.6%), 24/145 (16.6%), and 9/55 (16.4%), respectively, and the incidence of mucositis was 5 (5.7%), 4 (2.8%), and 0 (0%), respectively (33). It is obvious that these proportions could have largely occurred by chance (sampling variation): for example, if the true rate of diarrhea in CC subjects is indeed 20.6% (22/107) then the probability (28%) of observing 21-23/107 patients with diarrhea is not much different from the probability (27%) of observing 18-20/107 or the probability (30%) of observing 24-26/107 patients with such an event. By sheer chance, with comparable probability, one could have observed the incidence of 24.2% (instead of 20.6%) or 16.8% – which is almost identical as in TT patients (16.4%). In TT patients or in the case of mucositis, the fragility of the proportions is even greater: one could have observed one or two events more or less in any of the patient subsets (which would have been equally as probable as the observed numbers), and this would have given a completely different impression. Another problem with such samples is that it might be difficult to achieve conditional exchangeability between the exposed and control group on (even) the measured confounders. For example (33), univariate analysis did not find an association between the rs1128503 variant and the incidence of diarrhea (*P* = 0.676 by χ^2^ test) (33) or mucositis (*P* = 0.245) (33). However, the authors reported that in “multivariable models” (likely logistic regression models) the “effect” of genotype (2 levels, CC vs CT/TT) was – with adjustment for sex (2 levels), chemotherapy protocol (3 levels), line of therapy (2 levels), *UGT1A1* genotype (4 levels), and age (continuous) – “statistically significant,” ie, that the CT/TT genotypes had a “significantly lower” incidence of diarrhea (*P* = 0.014) and mucositis (*P* = 0.002) (effect measures, presumably odds ratios, were not reported) (33). This seems a rather unconvincing claim: namely, there were only 55 diarrhea events and 9 mucositis events (among 307 subjects, ie, the incidence of 17.9% and 2.9%, respectively) (33). Although there is no explicitly proven “rule of thumb” for determining the sample size for a logistic regression analysis (34), when the sample is modest (as in [33]), with a modest or low proportion of events (as in [33]), and with a small number of events per every “explanatory variable” in the model (eg, <10 in [33]), the typically employed maximum likelihood estimates of coefficients (odds ratios) are highly likely to be seriously inaccurate (34). Hence, the reported “significance” makes little sense, since it was likely biased and overtly model-dependent. Probably, other methods (weighting, matching) (35) would have been more appropriate, but even those will not “function” as desired in small samples. Proportions are particularly susceptible to fragility due to limited sample sizes and numbers of events, but continuous outcomes could also be by chance in small samples. In general, large effects reported from small studies are unlikely to be close to the true effects (36). Large effects observed in large(r) studies are less problematic even if not adjusted for potential confounders – it is less likely that they could be completely explained by sampling variability and/or residual confounding/bias (as argued by Cornfield in 1957 that smoking caused lung cancer) (37). Small(er) effects (and true effects of individual genetic variants are more likely to be closer to “small” than to “large”) (36) are always tricky for interpretation: they may indeed be accurate in top-quality observational studies (38), but sometimes they might be completely explained by even a small residual confounding (20).

It is commonly thought that pooling data across many small studies can resolve “their problems,” since the total number of included subjects could be large. However, a larger number of patients across a number of small studies does not correspond to one large study of the same size – a meta-analysis cannot amend the individual study biases and confounding, and by-chance results. This is additionally “complicated” by clinical heterogeneity and heterogeneity of estimates across studies. The observed “narrower confidence intervals” (precision) around a pooled estimate have nothing to do with its accuracy, and its meaning is typically misinterpreted, particularly in the case of observational studies (39). A meta-analysis is a potentially powerful tool, but there are many methodological “tricks of the trade” that need to be mastered (and are typically overlooked in most of the published meta-analyses) in order to produce meaningful results and interpret them accordingly (see [40] for a comprehensive guide). What is typically overlooked is the fact that candidate-gene Pgx studies are not randomized controlled trials (RCTs). They are observational studies; they generate estimates under different conditions and correspond to “non-randomized studies of interventions” – hence, this is how they should be meta-analyzed and interpreted. A recent comprehensive guide provides a step-by-step methodological roadmap for a meaningful conductance and interpretation of systematic reviews and meta-analyses of observational studies (41). Here, let me just list a few essential points: i) observational studies (vs RCTs) are (at least in part) susceptible to different types of biases (42); ii) different types of observational studies (cohort, case-control, or cross-sectional) are susceptible to different biases and “target” different estimands – they should be evaluated accordingly, and the results should not be pooled across different designs; iii) if data pooling is at all appropriate, the “object” of pooling should be adjusted effect estimates rather than “raw” counts (proportions) or means, or similar; iv) in terms of causality, the meta-analytical pooled estimates are informative only under the assumptions that (a) exposure was randomized within the levels of controlled variables (ie, in the case of observational studies – no uncontrolled confounding), (b) there was no uncontrolled measurement error, (c) there was no uncontrolled selection bias, and (d) the model on which the interval estimate was based was not misspecified – conditions that are practically never fully met in observational studies (39); v) in the observational setting, the fixed-effect estimates are almost never appropriate, while random-effects estimates only make sense if heterogeneity of true effects can be thoroughly investigated. Generally, unlike in the case of well-performed RCTs with results that are all “in the same direction,” the objective of a meta-analysis of observational studies should not be a single summary effect estimate but a search for sources of between-study heterogeneity (39,43,44).

**Figure 5:**
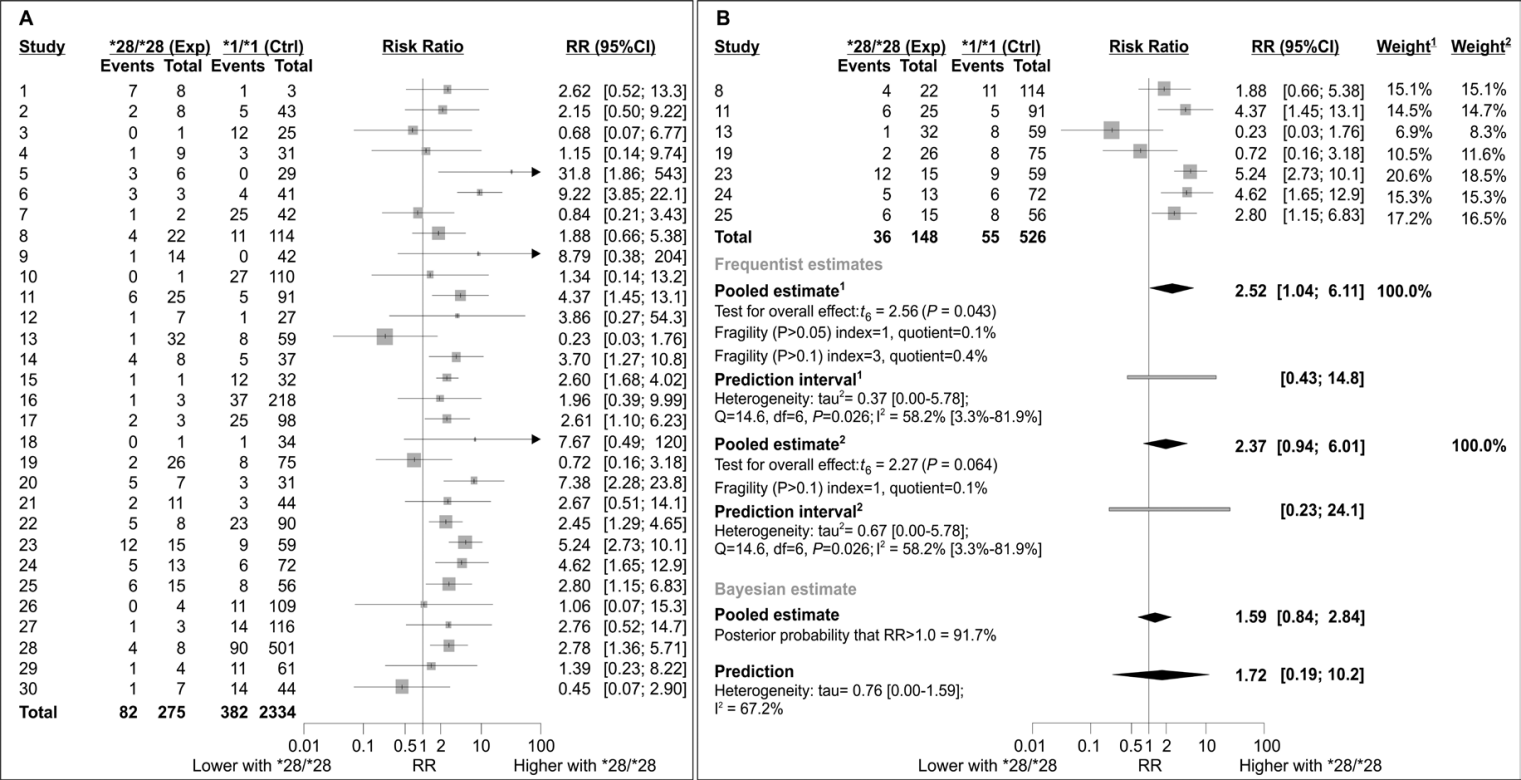
An example of a typical meta-analysis of candidate-gene pharmacogenomics/pharmacogenetics studies and illustration of the importance of heterogeneity and fragility. (**A**) Individual study results from a meta-analysis that aimed to assess whether carriers of the loss-of-function alleles (*28/*28, Exposed, Exp) in the gene encoding the metabolizing enzyme UGT1A1 differed from wild-type subjects (*1/*1, Controls, Ctrl) in the incidence of severe neutropenia during treatment with irinotecan-based protocols. It was one of the many studies consulted in the preparation of the recent pharmacogenetic recommendations related to treatment with irinotecan (44). Here, it is anonymized, just as are the primary studies that it included (enumerated 1-30), since it is substantially flawed for many reasons (some are outlined in the main text). Intentionally, no pooled estimate is provided, since very likely it would be misleading. (**B**) A summary of the re-analysis of some of the studies from part A. From the standpoint of estimation of the effect of *28/*28 genotype, it is equally as misleading as part A (for the same reasons). However, it was undertaken for a different purpose: it includes only the studies with (i) at least 10 “exposed” subjects and (ii) at least 5 events among controls to illustrate the importance of heterogeneity and fragility, even if one would assume that all primary studies were perfectly unconfounded/unbiased, and not “miniature” in size. Two frequentists analyses (random-effects with Mantel Haenszel method for relative risk) were done, yielding the “pooled estimate 1” (restricted maximum likelihood estimator of tau^2^) and the “pooled estimate 2” (Paul-Mandel estimator of tau^2^), both with Hartung-Knapp-Sidik-Jonkman correction for random effects. A Bayesian (random-effects) estimate was also generated, with a moderately informed skeptical normal prior for ln(RR) (N(0,0.355)), and a weakly informative half-Cauchy (0, 0.5) prior for tau.

To illustrate a prototypical example of a meta-analysis of candidate-gene Pgx studies (methodological and interpretational errors should be obvious based on the present elaboration and detailed methodological instructions [40,41]), the use is made of a generally accepted Pgx “genetic variant” with proven clinical relevance for the use of irinotecan in cancer patients (45). People carrying two loss-of-function alleles in the uridine diphosphate glucronosyltransferase 1A1 gene promoter (UGT1A1 *28/*28) are phenotypically defined as poor metabolizers (PM) of SN-38, the active metabolite of irinotecan. It has been recommended that PM (*28/*28) patients should be started at 70% lower irinotecan doses to minimize the risk of severe adverse events, particularly neutropenia (to a lesser extent diarrhea) (45). The recommendation is based on a large number of studies of different types, but the highest quality of evidence was assigned (45) to an analysis of 1200 colorectal cancer patients included in a previous large RCT comparing different treatment protocols (46). In this RCT, with adjustment for age, sex, body surface area, performance status, baseline neutrophil count (all also affecting the risk of severe neutropenia), in patients treated with 5-FU/leucovorin/irinotecan (but not in those treated with 5-FU/leucovorin), the *28/*28 genotype (vs *1/*1 or *1/*28, designated as normal or intermediate metabolizers, NM, IM) was associated with a higher risk of severe neutropenia (may be approximated [20] from the reported odds ratio as RR = 1.70, 95% 1.28-2.25). One of the meta-analyses of candidate-gene Pgx studies consulted in the preparation of the recommendations (45) (here, it remains anonymized, as well as the individual studies that it included) identified 30 prospective studies enrolling patients under different protocols with irinotecan. The meta-analysis assessed the incidence of severe neutropenia in *28/*28 (PM, “exposed”) vs *1/*1 patients (NM, “controls”) and reported a fixed-effect pooled OR = 3.50 (95%CI 2.25-5.50), which was interpreted as a “strong effect of PM phenotype.” Figure 5A presents all 30 individual studies (as displayed in the original meta-analysis) with individual relative risks (since deemed more intuitive than odds ratios) and intentionally shows no pooled estimate, as it would likely be misleading because: i) the approach to data was naïve, using simple “raw” proportions (as if from RCTs); ii) the number of “exposed” subjects was <10 in 21/30 studies, and ranged from 1 to 32, whereas the number of controls ranged from 3 to 501; iii) clinical heterogeneity was marked, whereas “numerical” heterogeneity is visible already on inspection (Figure 5A); iv) although the number of studies was large, individual study particulars were scarce, and no investigation of heterogeneity is possible. Figure 5B summarizes a re-analysis of some of the studies from the original meta-analysis. The re-analysis is – regarding the objective of “estimating the effect of the exposure of interest”, ie, PM phenotype – likely equally as misleading but serves a different purpose. It makes use only of the studies with >10 PM (“exposed”) subjects and with at least 5 events among NM control subjects and intends to illustrate the importance of heterogeneity and fragility, assuming that individual studies were completely unconfounded and unbiased, and not “miniature” in size. Two frequentist meta-analyses with different methods to estimate the across-study variance (although both estimates are questionable, since based on only 7 studies) – and a Bayesian meta-analysis were performed (Figure 5B): i) heterogeneity was huge in all analyses, and lower limits of the prediction intervals suggested that some true effects were in favor of the exposed (as much as 2.5-fold lower, 5-fold lower, and 5-fold lower risk in the exposed in the frequentist and Bayesian analyses, respectively), while some were pronouncedly in favor of controls (as much as 15-fold, 24-fold, and 10-fold higher risk in the exposed, respectively); ii) the estimates were extremely fragile – the frequentist “pooled estimate 1” suggested that the mean true effect was 2.52, with *P* = 0.048, but only 1 exposed or 1 control subject with an opposite outcome (non-event instead of event, or *vice-versa*) would have been needed to “push” the *P* value to >0.05, and 3 to “push it”>0.1 (fragility index in Figure 5B); iii) the number of studies was too low for exploration of heterogeneity, or for a meaningful analysis of sensitivity to unmeasured confounding (47). It might be argued that the original meta-analysis, despite all the limitations, pointed in the same direction as the final recommendations (45). However, in the case of Pgx, an accurate estimate of the size of an effect is crucial: it is the size of an effect (and not a mere fact of an effect) that determines whether it is plausible to consider a certain genetic variant as “critical” – one for which pre-emptive knowledge would be of practical relevance.

## Conclusion

Technological and conceptual developments in Pgx research are likely to enable improvements in individualized therapy that until recently appeared almost impossible. In this context, “classical” candidate-gene studies are commonly viewed as obsolete and outdated. In part, this is likely due to (too commonly) inadequate execution of such studies, in the sense of non-compliance with the well-established rules for their meaningful planning and conductance. The present considerations are intended to suggest that implementation of certain concepts established in analytical epidemiology may improve the validity and usefulness of estimates generated in candidate-gene Pgx studies, and in meta-analyses of such studies.
